# The Role of Web-Based Adaptive Choice-Based Conjoint Analysis Technology in Eliciting Patients’ Preferences for Osteoarthritis Treatment

**DOI:** 10.3390/ijerph20043364

**Published:** 2023-02-14

**Authors:** Basem Al-Omari, Joviana Farhat, Mujahed Shraim

**Affiliations:** 1 Department of Epidemiology and Population Health, College of Medicine and Health Sciences, Khalifa University, Abu Dhabi P.O. Box 127788, United Arab Emirates; 2 Faculty of Health and Life Sciences, The University of Northumbria, Benton, Newcastle upon Tyne NE7 7XA, UK; 3 Department of Public Health, College of Health Sciences, Qatar University, QU Health, Doha P.O. Box 2713, Qatar

**Keywords:** adaptive choice-based conjoint, web-based, conjoint analysis, patients’ preferences, osteoarthritis, shared decision making

## Abstract

Objective: To assess the feasibility of using adaptive choice-based conjoint (ACBC) analysis to elicit patients’ preferences for pharmacological treatment of osteoarthritis (OA), patients’ satisfaction with completing the ACBC questionnaire, and factors associated with questionnaire completion time. Methods: Adult patients aged 18 years and older with a medical diagnosis of OA, experiencing joint pain in the past 12 months, and living in the Northeast of England participated in the study. The participants completed a web-based ACBC questionnaire about their preferences regarding pharmaceutical treatment for OA using a touchscreen laptop independently, and accordingly, the questionnaire completion time was measured. Moreover, the participants completed a pen-and-paper feedback form about their experience in completing the ACBC questionnaire. Results: Twenty participants aged 40 years and older, 65% females, 75% had knee OA, and suffering from OA for more than 5 years participated in the study. About 60% of participants reported completing a computerized questionnaire in the past. About 85% of participants believed that the ACBC task helped them in making decisions regarding their OA medications, and 95% agreed or strongly agreed that they would be happy to complete a similar ACBC questionnaire in the future. The average questionnaire completion time was 16 min (range 10–24 min). The main factors associated with longer questionnaire completion time were older age, never using a computer in the past, and no previous experience in completing a questionnaire. Conclusions: The ACBC analysis is a feasible and efficient method to elicit patients’ preferences for pharmacological treatment of OA, which could be used in clinical settings to facilitate shared decision-making and patient-centered care. The ACBC questionnaire completion consumes a significantly longer time for elderly participants, who never used a computer, and never completed any questionnaire previously. Therefore, the contribution of patients and public involvement (PPI) group in the development of the ACBC questionnaire could facilitate participants’ understanding and satisfaction with the task. Future research including patients with different chronic conditions may provide more useful information about the efficiency of ACBC analysis in eliciting patients’ preferences for osteoarthritis treatment.

## 1. Introduction

Osteoarthritis (OA) is a chronic disabling condition that mainly affects elderly people and females, it still has no cure and is associated with functional limitation and reduced quality of life [1,2]. The management of OA aims to control the disease progress, reduce pain, and improve mobility and it includes pharmaceutical, non-pharmaceutical, and surgical treatment [3]. Pharmacological treatment for OA includes a wide range of analgesia and anti-inflammatory medications, such as acetaminophen, Nonsteroidal Anti-Inflammatory Drugs (NSAIDs), and opioids. In the United Kingdom (UK), many pain-relieving medications used for OA, such as paracetamol, and some types of NSAIDs are available without a doctor’s prescription. These medications are associated with several side effects, such as gastric ulcers, liver and kidney toxicity, and cardiovascular side effects [4,5]. If patients prefer these medications, they can buy and take them without their physicians’ knowledge or approval. Subsequently, patients may avoid the use of prescription medications, which will be wasted. The Department of Health estimates that unused medicines cost the NHS around £300 million every year due to patients’ incompliance with treatment [6]. In this case, patients may benefit or expose themselves to the risks of side effects from over-the-counter medications. Especially given that OA medications are associated with several side effects, such as gastric ulcers, liver and kidney toxicity, and cardiovascular side effects [4,5]. Therefore, the elicitation of patient preferences concerning the pharmacological treatment for OA is of particular importance, especially because treatment-based decisions are rarely urgent [7,8]. Furthermore, it is also emphasized that studying patients’ preferences for treatment options and considering which treatment factors are valued by patients may help in moving decision-making toward a patient-centered direction [9] along with improved patient satisfaction [10].

The number of studies exploring treatment-directed decisions in people with OA and trading off one treatment factor for another in their decision-making plan is still limited [11]. If clinicians are aware of patients’ preferences for pharmaceutical treatment for OA, they are likely to mutually agree on these treatment choices with patients. Consequently, this mutual agreement could minimize the risk of side effects of over-the-counter medications and reduce the wastage of prescribed medications. Several methods have been used to study patients’ preferences, such as traditional quantitative and qualitative methods. Qualitative and standard quantitative questionnaire methods have been used to study patients’ preferences regarding OA medication [11]. However, these methods have been used to obtain general insights about patients’ preferences, not examining the trade-offs that patients make between several treatment factors [12].

Conjoint analysis (CA) is a survey methodology that can be used to study preferences [13] and quantify the trade-offs made by patients when choosing between multiple treatment options [14]. Unlike traditional questionnaires, conjoint analysis poses several hypothetical scenarios, and asks patients to rate/rank them or choose their preferred scenario. The popularity of conjoint analysis (CA) methods has significantly increased in healthcare within the last two decades [15,16]. Healthcare professionals have been driving their efforts toward a patient-oriented profession to ameliorate patient compliance with treatment, prognosis, and well-being.

The denomination “conjoint” indicates that several factors can be “considered jointly” [17]. Therefore, CA allows people to choose between different hypothetical treatment scenarios rather than considering their characteristics individually. CA proposes people with ideas that closely resemble the decisions made in real life when choosing between options [18].

Adaptive choice-based conjoint (ACBC) analysis is the latest CA methodology derived from the choice-based conjoint (CBC) method used to understand consumers’ preferences for products [19,20]. The term “adaptive” means that respondents’ preferences are adjusted based on their selected choices of attributes and levels of a particular product [21]. Compared to the traditional CBC, the ACBC technique can easily handle a large number of attributes (>5 attributes) to ensure a full product profiling and could handle extreme response behavior that happens frequently with the CBC technique [22,23,24]. Another adaptive method is the Adaptive Conjoint Analysis (ACA), which was used to aid individually tailored treatment decision-making [25]. ACA has been increasingly applied in studies assessing patients’ preferences regarding medication treatment at a group level [26,27]. However, it is suggested that ACBC offers a more engaging experience for participants and a more accurate prediction of respondents’ choices and behavior by collecting more data from each respondent [21]. This comprehensive data collection allows ACBC to improve the estimation of utilities and real-world preferences’ expectations [28]. ACBC is usually associated with lower standard errors than the conventional choice-based CA [20], allowing a more accurate measurement of responses related to price when the cost is included as an attribute [21,22]. Taking into consideration that ACBC is a computer-based method [20], while the computational power, software development, and computer availability are increasing [29], it is expected that ACBC is gaining more popularity.

The ACBC was used in marketing research to elicit people’s preferences for products, such as electric vehicles and food choices [21,30], and assess people’s willingness to pay for these products [31]. ACBC was also applied in educational settings, such as identifying participants’ preferences for components of a cyberbullying prevention program [32]. In social life, the ACBC was used to assess people’s social needs [33]. Certainly, ACBC tends to formulate a more user-centered survey process than conventional methods [34]. Therefore, it is being implemented in healthcare settings, such as hospitals [35], breast cancer, and OA clinics, to elicit patients’ preferences for treatments or settings [36,37].

Despite its advantages, the ACBC method takes a longer completion time than the conventional CBC as it integrates a higher number of attributes and levels [38]. In some cases, respondents might be exhausted resulting in incomplete data [39]. It is still unclear if the ACBC method has an acceptance by patients with OA, and if it takes a longer time to complete. This can impose an important consideration in the case of OA where patients are mostly elderly; therefore, it is expected that completing an ACBC questionnaire might be challenging for them. A feasibility study by Al-Omari and colleagues in 2015 demonstrated that the ACBC is a feasible method to elicit patients’ preferences for pharmacological treatment of OA. Consequently, the survey completion time did not differ substantially between participants who use a computer daily and participants who did not use or have a computer [40]. However, Al-Omari and colleagues’ study included a small sample of the Research Users Group (RUG) who have experience in participating in research and did not assess survey completion time. Although ACBC has been applied in multiple healthcare settings, there is limited evidence regarding whether or not ACBC methodology would be feasible for assessing attributes associated with OA medications due to their complexity and the various levels of risks involved [16,37,40]. Therefore, this study is assessing the feasibility of using the ACBC to elicit patients’ preferences for pharmacological treatment of OA, patients’ satisfaction with completing the ACBC questionnaire, and factors associated with questionnaire completion time using a public sample of patients with OA.

## 2. Materials and Methods

### 2.1. Population, Setting, and Study Design

Study participants were from Newcastle-upon-Tyne and surrounding areas in the northeast of England. Posters, emails, and newsletters about the study were published via Healthwatch Newcastle, Healthwatch North-East, and employees and students at the faculty of health and life sciences at Northumbria University. Healthwatch Newcastle is one of the 152 Healthwatch that operate in each local authority area in England [41], and they are authorized by the Health and Social Care Act 2012 [42]. Healthwatch is independent of healthcare providers and aspires to assist children, young people, and adults to be able to evaluate health services, and to include people who sometimes struggle to be heard [41].

Adult patients above the age of 18 years old with a self-reported medical diagnosis of OA, experiencing joint pain in the past 12 months, and residing in Newcastle upon Tyne and surrounding areas in the Northeast of England were eligible of being part of the study. Patients were excluded from the study if they (1) are not experiencing joint pain, and (2) have other illnesses that may be associated with or cause their joint pain, such as rheumatoid arthritis or osteoporosis. Patients who showed interest in participating in the study were contacted by phone and/or email to determine their eligibility for inclusion. Study participants were visited at home to complete the online ACBC questionnaire. The completion time for the ACBC task was recorded, then a feedback form regarding patients’ experiences with completing the ACBC questionnaire was completed by the patients.

### 2.2. The ACBC Questionnaire Development

The web-based ACBC questionnaire was built using Sawtooth Software Lighthouse studio, version 8.2.4. The ACBC design integrates three major sections: build-your-own (BYO) configurators, a screening section, and choice-based tasks [43]. In the BYO section, a list of attributes and levels is presented to allow participants to choose their most preferred level for each attribute. Based on each respondent’s BYO answers, a collection of treatments’ characteristics is generated. Then, in the screening section, the respondents are presented with several hypothetical scenarios and asked to indicate if accepting each scenario is “a possibility” or “not a possibility”. During the screening section, the scenarios are further narrowed down by offering respondents “Must have” and “Unacceptable” options to select the levels that they will not give away or trade-off and others that are completely rejected, respectively. Finally, the choice tasks section allows respondents to select their preferred scenario from several scenarios within their consideration set using a tournament format. The process of developing the ACBC questionnaire was the same as that previously implemented by Al-Omari and colleagues’ studies [37,40,43,44] and included 9 attributes and 31 levels (see Appendix A for a full list of attributes and levels). An example of the full ACBC questionnaire is found in a previous paper by Al-Omari and colleagues [37]. The wording and formatting of the ACBC attributes, levels, and sentences in the scenarios were also consistent with the feedback of the Research Users Group (RUG). RUG members are real patients present in society and suffering from OA. In the previous ACBC feasibility study, RUG participants contributed to the design, content development, and direct usage of ACBC [40]. Consequently, the active involvement of the patients and public involvement (PPI) in research constitutes a good indicator of a more relevant and better-designed study with clearer results and updated evidence [43].

### 2.3. Data Collection

Personal information was not requested in the ACBC questionnaire, and all participants were anonymous. Each respondent had a unique ID number and a password to complete the questionnaire. The identification (ID) numbers and passwords were given to the respondents before completing the questionnaire on a random basis and were not personally identifiable. The data to match the respondents’ ID numbers with their identity (name, phone number, and email address) were recorded on password-protected files that were accessible only by the lead researcher (BA).

The ACBC questionnaire was created on a password-protected laptop, then hosted on a web server by the Technical Architect Department at Northumbria University (Appendix B-Table A2 shows the web server specifications). All actions complied with Northumbria University guidelines for the storage of sensitive and confidential data on laptops (2014 version).

The conceptual infrastructure model of the server was developed by the cloud solutions and infrastructure engineer at the Technical Architect Department at Northumbria University, (Appendix B-Figure A1 shows the created service on the webserver). The server was also used as a host for the collected data. Participants were able to access and complete the ACBC questionnaire from any device connected to an internet source. Automatic daily backup of the server was instructed to minimize the risk of losing the data. Furthermore, a daily manual backup of the data to the administrator’s laptop was implemented by the lead researcher (BA).

The data were collected in two stages. In stage 1, participants were provided with a touchscreen laptop that has the Uniform Resource Locator (URL) address of the online ACBC questionnaire and their unique username and password. Then, the participants were asked to independently complete the web-based ACBC questionnaire concerning their preferences regarding the pharmaceutical treatment of OA. The length of time required by each participant to complete the ACBC questionnaire was recorded using the laptop system clock and a stopwatch with the researcher to ensure accuracy. The following variables were collected to investigate any association with questionnaire completion time: age group (40–59, 60–79, and >79 years), gender, duration of OA (<5, 5–10, and >10 years), pain level (not at all, a little bit, moderate, quite a bit, and extremely), frequency of computer use per week (every day, a few times, very rarely, and do not use a computer), completion of a computerized questionnaire in the past (yes, no, and do not remember), and completion of a pen and paper questionnaire in the past (yes, no, and do not remember). In stage 2, each participant was asked to independently complete a feedback form about his/her experience in completing the ACBC questionnaire. The practicality of ACBC was measured through the feedback form including tick-box questions and 5 Likert items ranging from strongly agree to strongly disagree. This feedback form included the following questions: “I found the questionnaire easy to read, I found the questionnaire easy to understand, I felt that the questionnaire was adjusting the questions according to my previous answers, completing the questionnaire helped me in making a decision about my preferences, I enjoyed completing the questionnaire, I would be happy to complete a similar computerized questionnaire in the future.”

### 2.4. Data Analysis

Descriptive statistics were used to summarize categorical variables using frequencies and percentages and continuous variables using the mean and the standard deviation (SD). As the questionnaire completion time in minutes is a continuous variable, bivariable linear regression was used to examine the relationship between predictor variables and questionnaire completion time, including age group, gender, computer, or laptop use frequency, completion of computerized questionnaires status, completion of pen/paper questionnaires, easiness of reading the questionnaire, and easiness of understanding the questionnaire. A *p*-value of less than 0.05 was considered statistically significant. SPSS computer program (version 24.0) (IBM, New York, NY, USA) was used to analyze the data.

## 3. Results

### 3.1. Participants’ Characteristics

Twenty adult individuals diagnosed with OA and reporting joint pain within the last 12 months participated in the study and completed both the online ACBC questionnaire and the pen-and-paper feedback form. Approximately 65% of the participants were females. About 25%, 50%, and 25% were aged 40–59, 60–79, and >79 years, respectively. Around 80% reported having OA for more than five years. Approximately 95% of the participants reported that joint pain affected their normal life ranging from “a little bit” (20%) to “extremely” (10%). Most participants (75%) suffered from knee OA and only 5% reported having foot or hand OA. Most participants (90%) reported using paracetamol, while none reported using capsaicin, meloxicam, or fentanyl for the treatment of OA. About 20% and 25% of participants indicated that they use the computer very rarely and do not use a computer, respectively. About 60% and 70% of participants reported completing computerized and pen and paper questionnaires in the past, respectively. Table 1 shows the participants’ characteristics.

### 3.2. Participants’ Feedback about the ACBC Questionnaire

All participants agreed or strongly agreed that the ACBC questionnaire was easy to read and easy to understand. All the participants have also agreed or strongly agreed that the ACBC questionnaire was adjusting the questions according to their previous answers. The majority of participants (90%) enjoyed completing the ACBC and 85% of participants believed that the ACBC task helped them in making decisions regarding their OA medications. The vast majority (95%) agreed or strongly agreed that they would be happy to complete a similar ACBC questionnaire in the future. None of the participants disagreed or strongly disagreed with any of the statements in the feedback form. Table 2 shows participants’ feedback.

### 3.3. Questionnaire Completion Time

**Figure 1:**
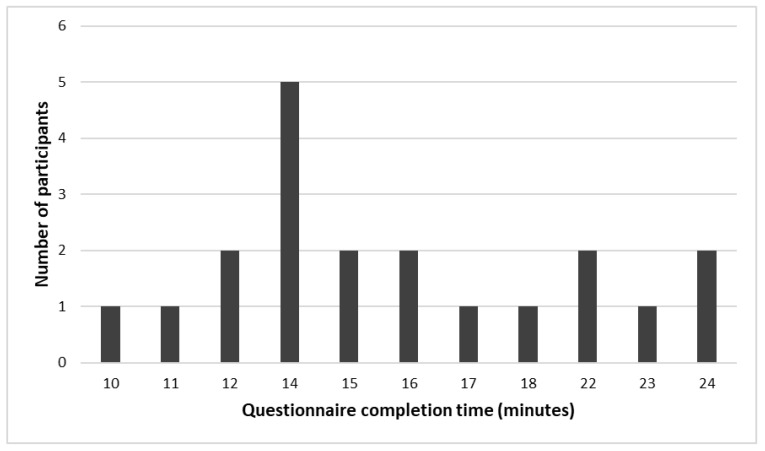
Distribution of time spent by participants completing the questionnaire.

Questionnaire completion time ranged between 10 min to 24 min, with an average completion time of 16 min. The participants had diverse computer skills ranging from people who did not use computers before (five participants (25%)) to those who reported using the computer daily (seven participants (35%)). The five participants who did not have a computer completed the questionnaire in 14, 16, 17, 23, and 24 min. About 40% and 30% of participants reported no history of completing computerized and pen and paper questionnaires, respectively. Figure 1 shows the distribution of questionnaire completion time.

### 3.4. Factor Associated with the Questionnaire Completion Time

Age group, computer use frequency, and previous completion of computerized or pen-and-paper questionnaires were the only variables showing statistically significant associations with questionnaire completion time. On average, participants aged 60–79 years spent 5.3 more minutes completing the questionnaire than those aged 40–59 years (95% confidence interval (CI), 1.4, 9.2). Similarly, as compared to participants aged 40–59 years, participants aged >79 years spent 4.4 more minutes completing the questionnaire, but this was not statistically significant (95% CI −0.2, 9.0). Participants who never used a computer in the past spent 4.9 more minutes completing the questionnaire than participants who reported using a computer every day (95% CI 0.6, 9.3). Participants who completed computerized and pen/paper questionnaires in the past spent less time completing the questionnaire than those who did not by 4.0 min (95% CI 0.6, 7.4) and 6.2 min (95% CI 3.1, 9.2), respectively. Table 3 presents the crude relationships between included variables and questionnaire completion time.

## 4. Discussion

This study assessed the feasibility of using the ACBC questionnaire for eliciting patients’ preferences regarding OA pharmacological treatment and evaluated patients’ satisfaction following the completion of the ACBC questionnaire. Moreover, this study determined the factors associated with ACBC questionnaire completion time.

In this study, most of the participants were females, over 60 years of age, suffering from knee OA, and had OA for more than 10 years. This is consistent with the previous literature reporting that OA mainly affects females and elderly people, and the knee is the most commonly affected joint [1,45,46]. Furthermore, most participants were exposed to pain moderately to extremely affecting their quality of life and have taken multiple medications of various levels of benefits and adverse events. This may suggest that the enrolled participants have a good understanding of their needs, preferences, and realistic expectations while choosing between OA treatment alternatives. This is also harmonious with previous studies reporting that OA patients usually acquire proper knowledge through their lived experience of managing a chronic condition [1], and from using various health services, which can facilitate a clear understanding of their healthcare needs and preferences [47].

All participants confirmed that the ACBC questionnaire was clear and easy to understand. Overall, patients were satisfied with the design and content of the ACBC questionnaire. This may be associated with working with the RUG during the development of attributes, levels, and ACBC questionnaire, which was followed by comprehensive developmental phases and pilots [40,43,44]. A previous study recommended integrating easily understandable, credible, and realistic attributes in a conjoint study to better understand patients’ preferences regarding the pharmaceutical treatment of OA [43]. It was also suggested that patients’ participation in research development increases the relevance and practical use of research outcomes [48]. In this ACBC study, special considerations were given to the wording of the attributes and levels to make them short, clear, simple, and user-friendly. Another reason that may have contributed to the clarity of this ACBC questionnaire in this study was providing clear instructions on how to complete the ACBC task on the introductory page of the questionnaire. This, in turn, reduced the number of words that had to be used to describe the attributes and levels. Therefore, the participants did not feel overwhelmed by the amount of information on each screen. Furthermore, the ACBC questionnaire was entitled “Your views on osteoarthritis treatment” which may provide a sense of “ownership” making the participants feel that the researchers are interested in the patients’ views and not testing them.

In this study, all participants reported that the ACBC questionnaire was enjoyable to complete, adjusting the new questions according to their previous answers, and its completion helped them decide about their preferences. These findings support the suggestion that ACBC can engage the respondent with the task, and, subsequently, they provide answers that represent their preferences compared to other respondents who used different CA techniques [20,38,49,50]. A previous study investigated the ACBC completion time in the OA setting indicated a mean completion time of 24 min [40], compared to the mean time of 16 min in our study. The longer time in Al-Omari and colleagues’ study with the RUG could be due to the participants being instructed to critically investigate the ACBC task while completing it questionnaire [40]. Conversely, in this study, the participants were instructed to complete the task solely for preference elicitation.

The results of the bivariable linear regression suggested that the statistically significant attribute that may have an impact on extending the ACBC questionnaire completion time are age group (60–79 years old), computer use frequency (if the patient never used the computer), and previous completion of computerized or pen and paper questionnaires (if the patient never completed a questionnaire previously). To date, there are very few studies investigating the feasibility of using ACBC and other CA techniques with OA patients. A previous similar study by Rochon and colleagues using the ACA technique with OA patients showed that the ACA task presented a challenge to elderly OA participants due to their relatively low level of computer comfort [51]. The main difference between ACBC and ACA is that ACBC is choice-based while ACA is rating/ranking. ACA shares some technical features with ACBC such as being a CA technique that is adaptive. The participants in Rochon and colleagues reported not being comfortable adapting to the offered preferences during the ACA task [51]. To improve participant understanding and engagement in the task, Rochon and colleagues recommended choosing the software and presenting the treatment attributes with care, and educating participants about the task’s content and what to expect [51]. Therefore, it is reasonable to suggest that the participants’ engagement and satisfaction with the ACBC task were due to the careful and comprehensive steps that were taken during the development of the ACBC questionnaire with the support of the RUG.

### 4.1. Strengths and Limitations

To the best of our knowledge, this is the first study to report the association between patients’ characteristics and questionnaire completion time. Furthermore, this ACBC study addressed the limitation of the previous feasibility study of using ACBC with OA patients [40] by increasing the number of attributes and levels and the number of participants in the study. In this study, 20 participants were asked to complete the questionnaire independently without asking any questions, and no technical or content understanding problems were reported by the participants. The main limitation of this study is including a small sample size from one region. However, for the ACBC method, this sample size is generally sufficient for eliciting patients’ preferences, which promotes the individualization of patients’ preferences for better-shared decision-making and patient-centered care. Furthermore, this study investigated the feasibility of using the ACBC with OA patients, and the sample size is larger than the previous study investigating this feasibility [40]. Another limitation is that the participants completed the ACBC feedback form in the presence of the lead researcher, which might have introduced social desirability bias.

### 4.2. Clinical and Research Implications

Clinicians usually schedule a ten to fifteen minutes routine appointments for each patient consultation. During this relatively short consultation time, clinicians may find it difficult to judge patients’ preferences concerning OA treatment [52]. Therefore, tools that elicit patients’ preferences and improve the shared decision-making process are important [52,53]. One important clinical implication is that the ACBC technique appears to be a feasible method to elicit patients’ preferences regarding OA treatment and subsequently facilitates the shared decision-making process. This could be a major contribution toward the time management of clinicians in primary care and outpatient settings. For example, the patient could complete the ACBC questionnaire before the consultation and his/her preferences are made available to the clinician. Then, during the consultation, the clinician could discuss the patient’s preferences and prescribe the relevant medication accordingly. However, future research needs to examine the practicality of this in clinical settings.

## 5. Conclusions

In conclusion, this study confirmed the ACBC’s ability to offer direct and easily understandable content to participants in order to elicit their preferences regarding OA pharmacological treatment. Moreover, this feasibility study supported the ability of ACBC to provide a more relevant and engaging experience for participants with OA. This study indicated that the ACBC questionnaire completion consumes a significantly longer time for elderly participants, who never used the computer, and never completed any questionnaire previously. We suggest that researchers undertaking an ACBC study should carefully select the attributes and levels and explain the task to the participants. Correspondingly, engaging a PPI group in the development of the ACBC questionnaire facilitates participants’ understanding and satisfaction with the task. As ACBC has the potential to fit into routine primary care and outpatients’ practice when completed before the consultation, the ACBC questionnaire can be a suitable tool to elicit older patients’ preferences regarding the pharmaceutical treatment of OA, contribute to doctors’ understanding of patient’s preferences, and facilitate an informed shared decision-making process.

## Figures and Tables

**Table 1:** Characteristics of participants (*n* = 20).

Variable	Number (%)
Age group (years)	
40–59	5 (25)
60–79	10 (50)
>79	5 (25)
**Gender**	
Female	13 (65)
Male	7 (35)
**Years suffering from OA**	
<5	3 (15)
5–10	6 (30)
>10	10 (50)
Do not know	1 (5)
**Pain interference with normal life**	
Not at all	1 (5)
A little bit	4 (20)
Moderately	7 (35)
Quite a bit	6 (30)
Extremely	2 (10)
Site of OA *	
Hip	7 (35)
Knee	15 (75)
Back and Neck	3 (15)
Foot or hand	1 (5)
Other	2 (10)
Medication *	
Paracetamol	18 (90)
Capsaicin	0 (0)
Glucosamine	8 (40)
Diclofenac	9 (45)
Etodolac	2 (10)
Ibuprofen	10 (50)
Meloxicam	0 (0)
Naproxen	1 (5)
Codeine or Dihydrocodeine	11 (55)
Tramadol	6 (30)
Fentanyl	0 (0)
Morphine	2 (10)
Oxycodone	2 (10)
Others	3 (15)
**Computer usage per week**	
Every day	7 (35)
A few times every week	4 (20)
Very rarely	4 (20)
Do not use a PC	5 (25)
**Completion of a computerized questionnaire in the past**	
Yes	12 (60)
No	8 (40)
**Completion of pen and paper questionnaire in the past**	
Yes	14 (70)
No	6 (30)

* Total number will add to more than 20 as the participants were allowed to select more than one answer if they have multiple joints osteoarthritis.

**Table 2:** Participants’ feedback regarding the ACBC questionnaire.

Feedback Statement	Frequency				
	Strongly Agree	Agree	Neither Agree Nor Disagree	Disagree	Strongly Disagree
I found the questionnaire easy to read	15	5	0	0	0
I found the questionnaire easy to understand	14	6	0	0	0
I felt that the questionnaire was adjusting the questions according to my previous answers	14	6	0	0	0
I enjoyed completing the questionnaire	13	5	2	0	0
Completing the questionnaire helped me in making a decision about my preferences	11	6	3	0	0
I would be happy to complete a similar computerized questionnaire in the future	12	7	1	0	0

**Table 3:** Crude relationships between predictor variables and questionnaire completion time.

Variable	B	SE	95% CIs	*p*-Value
Gender				
Female (Ref)				
Male	−0.5	1.5	−4.1, −3.2	0.763
Age group (years)				
40–59 (Ref)				
60–79	5.3	2.0	1.4, 9.2	0.008
>79	4.4	2.3	−0.2, 9.0	0.058
Number of years suffering from OA				
>10 years (Ref)				
5–10 years	3.1	2.0	−0.8, 7.1	0.122
<5 years	2.3	2.6	−2.8, 7.4	0.374
Pain interference with daily life				
Not at all to a little bit (Ref)				
Moderately	−0.4	2.4	−5.0, 4.2	0.865
Quite a bit to extremely	2.7	2.3	−1.8, 7.2	0.235
Computer/laptop use frequency per week				
Every day (Ref)				
Few times	2.9	2.4	−1.8, 7.6	0.225
Very rarely	3.4	2.4	−1.3, 8.1	0.155
Never	4.9	2.2	0.6, 9.3	0.026
Completion of computerized questionnaires				
No (Ref)				
Yes	−4.0	1.7	−7.4, −0.6	0.021
Completion of pen/paper questionnaire				
No (Ref)				
Yes	−6.2	1.6	−9.2, −3.1	<0.001
I found the questionnaire easy to read				
Strongly agree (Ref)				
Agree	0.1	2.2	−4.3, 4.43	0.976
I found the questionnaire easy to understand				
Strongly agree (Ref)				
Agree	−1.5	2.1	−5.5, −2.6	0.482

Ref = Reference Category; B = Regression Coefficient for questionnaire completion time; SE = Standard Error; CI = Confidence interval.

**Figure A1:**
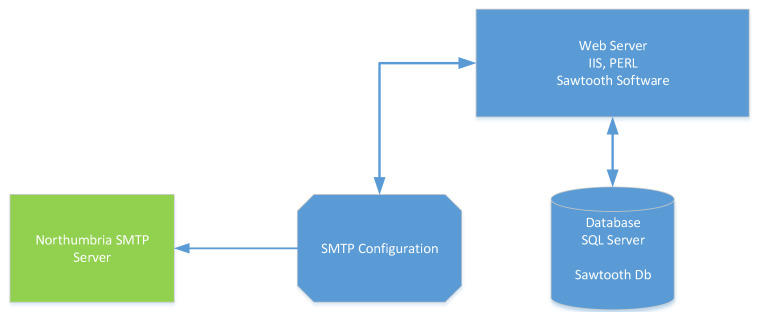
Overview of entire server design. Green Shows existing service to interface with, and blue shows the infrastructure to be created; Simple Mail Transfer Protocol (SMTP); Internet Information Services (IIS); Practical Extraction and Reporting Language (PERL); Structured Query Language (SQL); Database (Db).

## Data Availability

Not applicable.

## Appendix A

**Table A1:** Attributes and levels used in the ACBC study.

Attribute	Levels
Availability	Prescription drug
	Over-the-counter drug
	Internet purchase drug
Way of taking the medication	Cream/Gel
	Oral
Frequency	Once a day
	Twice a day
	3–4 times a day
	As needed
How much you would expect mobility improvement	Expect 25% mobility improvement
	Expect 50% mobility improvement
	Expect 75% mobility improvement
How much you would expect pain reduction	Expect 25% pain reduction
	Expect 50% pain reduction
	Expect 75% pain reduction
Risk of gastric ulcer	No risk of gastric ulcer
	Low risk of gastric ulcer
	Moderate risk of gastric ulcer
	High risk of gastric ulcer
Risk of addiction	No risk of addiction
	Low risk of addiction
	Moderate risk of addiction
	High risk of addiction
Risk of kidney and liver impairment	No risk of kidney and liver impairment
	Low risk of kidney and liver impairment
	Moderate risk of kidney and liver impairment
	High risk of kidney and liver impairment
Risk of heart attacks and strokes	No risk of heart attacks and strokes
	Low risk of heart attacks and strokes
	Moderate risk of heart attacks and strokes
	High risk of heart attacks and strokes

## Appendix B

**Table A2:** Server production specifications.

Processors	Quad Core Processor (or equivalent)
Operating System	Windows Server 2012 R2 Standard 64-bit
Network Speed	Higher than 100 Mbps
Network LAN and IP Address:	Provided by Server and Storage
Port Assignments	Defaults—Assigned by the relevant specialist
Installed Memory (RAM)	8.00 GB
Hard Disk	100 GB (System) 100 GB (Apps)
Disk Partitions—Drive Mappings	Defaults—For Applications
Additional software	IIS and PERL

## References

[1] NICE Overview|Osteoarthritis: Care and Management|Guidance|NICE. https://www.nice.org.uk/guidance/cg177.

[2] Li A., Zhang Y., Lao L., Xin J., Ren K., Berman B.M., Zhang R.-X. (2011). Serotonin receptor 2A/C is involved in electroacupuncture inhibition of pain in an osteoarthritis rat model. Evid. Based Complement. Alternat. Med..

[3] Al-Omari B., Hill B. (2020). Nursing people with osteoarthritis. Br. J. Nurs..

[4] Koeberle A., Werz O. (2009). Inhibitors of the microsomal prostaglandin E(2) synthase-1 as alternative to non steroidal anti-inflammatory drugs (NSAIDs)—A critical review. Curr. Med. Chem..

[5] Bindu S., Mazumder S., Bandyopadhyay U. (2020). Non-steroidal anti-inflammatory drugs (NSAIDs) and organ damage: A current perspective. Biochem. Pharmacol..

[6] Department of Health and Social Care Action on Medicine Wastage and Improving Medicine Use. https://www.gov.uk/government/news/action-on-medicine-wastage-and-improving-medicine-use.

[7] Fraenkel L., Bogardus S.T., Concato J., Wittink D.R. (2004). Treatment options in knee osteoarthritis: The patient’s perspective. Arch. Intern. Med..

[8] Hiligsmann M., Pinto D., Dennison E., Al-Daghri N., Beaudart C., Branco J., Bruyère O., Conaghan P.G., Cooper C., Herrero-Beaumont G. (2019). Patients’ preferences for osteoarthritis treatment: The value of stated-preference studies. Aging Clin. Exp. Res..

[9] Say R.E., Thomson R. (2003). The importance of patient preferences in treatment decisions―Challenges for doctors. BMJ.

[10] Saultz J.W., Albedaiwi W. (2004). Interpersonal continuity of care and patient satisfaction: A critical review. Ann. Fam. Med..

[11] Laba T.-L., Brien J., Fransen M., Jan S. (2013). Patient preferences for adherence to treatment for osteoarthritis: The MEdication Decisions in Osteoarthritis Study (MEDOS). BMC Musculoskelet. Disord..

[12] Hauber A.B., Arden N.K., Mohamed A.F., Johnson F.R., Peloso P.M., Watson D.J., Mavros P., Gammaitoni A., Sen S.S., Taylor S.D. (2013). A discrete-choice experiment of United Kingdom patients’ willingness to risk adverse events for improved function and pain control in osteoarthritis. Osteoarthr. Cartil..

[13] Wong D.W., Fong C., Da Silva C.E., Lam C.S., Miller S.M. (2004). Rehabilitation Counseling Students’ Attitudes Toward People with Disabilities in Three Social Contexts. Rehabil. Couns. Bull..

[14] Lamiraud K., Geoffard P.-Y. (2007). Therapeutic non-adherence: A rational behavior revealing patient preferences?. Health Econ..

[15] Al-Omari B., Farhat J., Ershaid M. (2022). Conjoint analysis: A research method to study patients’ preferences and personalize care. J. Pers. Med..

[16] Al-Omari B., McMeekin P., Bate A. (2021). Systematic review of studies using conjoint analysis techniques to investigate patients’ preferences regarding osteoarthritis treatment. Patient Prefer. Adherence.

[17] Johnson R.M. (1974). Trade-Off Analysis of Consumer Values. J. Mark. Res..

[18] Byrne M.M., Souchek J., Richardson M., Suarez-Almazor M. (2006). Racial/ethnic differences in preferences for total knee replacement surgery. J. Clin. Epidemiol..

[19] Rao V.R. (2014). Applied Conjoint Analysis.

[20] Cunningham C.E., Deal K., Chen Y. (2010). Adaptive choice-based conjoint analysis: A new patient-centered approach to the assessment of health service preferences. Patient.

[21] Jervis S.M., Ennis J.M., Drake M.A. (2012). A Comparison of Adaptive Choice-Based Conjoint and Choice-Based Conjoint to Determine Key Choice Attributes of Sour Cream with Limited Sample Size. J. Sens. Stud..

[22] Gensler S., Hinz O., Skiera B., Theysohn S. (2012). Willingness-to-pay estimation with choice-based conjoint analysis: Addressing extreme response behavior with individually adapted designs. Eur. J. Oper. Res..

[23] Eggers F., Sattler H. (2011). Preference Measurement with Conjoint Analysis. Overview of State-of-the-Art Approaches and Recent Developments. GfK Mark. Intell. Rev..

[24] Brand B.M., Baier D. (2020). Adaptive CBC: Are the Benefits Justifying its Additional Efforts Compared to CBC?. Arch. Data Sci. Ser. A.

[25] Pieterse A.H., Berkers F., Baas-Thijssen M.C.M., Marijnen C.A.M., Stiggelbout A.M. (2010). Adaptive Conjoint Analysis as individual preference assessment tool: Feasibility through the internet and reliability of preferences. Patient Educ. Couns..

[26] Beusterien K.M., Dziekan K., Schrader S., Flood E., Flood R., Shearer A., Davis E.A. (2007). Patient preferences among third agent HIV medications: A US and German perspective. AIDS Care.

[27] Fraenkel L., Suter L., Cunningham C.E., Hawker G. (2014). Understanding preferences for disease-modifying drugs in osteoarthritis. Arthritis Care Res..

[28] Yu J., Goos P., Vandebroek M. (2011). Individually adapted sequential Bayesian conjoint-choice designs in the presence of consumer heterogeneity. Int. J. Res. Mark..

[29] Gustafsson A., Herrmann A., Huber F. (2000). Conjoint Measurement.

[30] Shin H.-S., Farkas Z.A., Nickkar A., Noyce D.A. (2019). An analysis of attributes of electric vehicle owners’ travel and purchasing behavior: The case of maryland. Proceedings of the International Conference on Transportation and Development 2019.

[31] Sichtmann C., Wilken R., Diamantopoulos A. (2011). Estimating Willingness-to-pay with Choice-based Conjoint Analysis-Can Consumer Characteristics Explain Variations in Accuracy?. Br. J. Manag..

[32] Cunningham C.E., Chen Y., Vaillancourt T., Rimas H., Deal K., Cunningham L.J., Ratcliffe J. (2015). Modeling the anti-cyberbullying preferences of university students: Adaptive choice-based conjoint analysis. Aggress. Behav..

[33] Veitch J., Ball K., Rivera E., Loh V., Deforche B., Timperio A. (2021). Understanding children’s preference for park features that encourage physical activity: An adaptive choice based conjoint analysis. Int. J. Behav. Nutr. Phys. Act..

[34] Louviere J.J., Street D., Burgess L., Wasi N., Islam T., Marley A.A.J. (2008). Modeling the choices of individual decision-makers by combining efficient choice experiment designs with extra preference information. J. Choice Model..

[35] de Groot I.B., Otten W., Smeets H.J., Marang-van de Mheen P.J. (2011). CHOICE-2 study group Is the impact of hospital performance data greater in patients who have compared hospitals?. BMC Health Serv. Res..

[36] Reinisch M., Marschner N., Otto T., Korfel A., Stoffregen C., Wöckel A. (2021). Patient Preferences: Results of a German Adaptive Choice-Based Conjoint Analysis (Market Research Study Sponsored by Eli Lilly and Company) in Patients on Palliative Treatment for Advanced Breast Cancer. Breast Care.

[37] Al-Omari B., McMeekin P. (2020). Patients’ Preferences Regarding Osteoarthritis Medications: An Adaptive Choice-Based Conjoint Analysis Study. Patient Prefer. Adherence.

[38] Orme B. (2009). Fine-Tuning CBC and Adaptive CBC Questionnaires. https://sawtoothsoftware.com/resources/technical-papers/fine-tuning-cbc-and-adaptive-cbc-questionnaires.

[39] Webb E.J.D., Meads D., Eskyte I., King N., Dracup N., Chataway J., Ford H.L., Marti J., Pavitt S.H., Schmierer K. (2018). A Systematic Review of Discrete-Choice Experiments and Conjoint Analysis Studies in People with Multiple Sclerosis. Patient.

[40] Al-Omari B., Sim J., Croft P., Frisher M. (2015). Patient preferences for the pharmacological treatment of osteoarthritis: A feasibility study using adaptive choice-based conjoint analysis (acbca). Eur. J. Pers. Cent. Healthc..

[41] About Us-Healthwatch Newcastle. https://www.healthwatchnewcastle.org.uk/about-us/.

[42] Health and Social Care Act 2012. https://www.legislation.gov.uk/ukpga/2012/7/contents/enacted.

[43] Al-Omari B. (2017). Patient preferences for the pharmacological treatment of osteoarthritis using adaptive choice-based conjoint (ACBC) analysis: A pilot study. Eur. J. Pers. Cent. Healthc..

[44] Al-Omari B., Sim J., Croft P., Frisher M. (2017). Generating Individual Patient Preferences for the Treatment of Osteoarthritis Using Adaptive Choice-Based Conjoint (ACBC) Analysis. Rheumatol. Ther..

[45] Hsu H., Siwiec R.M. (2022). Knee Osteoarthritis. StatPearls.

[46] Liu C., Du Z., Zhou Q., Hu B., Li Z., Yu L., Xu T., Fan X., Yang J., Li J. (2014). Microscopic examination of intracellular organisms in bronchoalveolar lavage fluid for the diagnosis of ventilator-associated pneumonia: A prospective multi-center study. Chin. Med. J..

[47] Song H.J., Dennis S., Levesque J.-F., Harris M.F. (2020). What do consumers with chronic conditions expect from their interactions with general practitioners? A qualitative study of Australian consumer and provider perspectives. Health Expect..

[48] de Wit M.P.T., Elberse J.E., Broerse J.E.W., Abma T.A. (2015). Do not forget the professional--the value of the FIRST model for guiding the structural involvement of patients in rheumatology research. Health Expect..

[49] Bailey C.M. New Adaptive Choice-Based Conjoint Technique Shows Promise|GreenBook|GreenBook.org. https://www.greenbook.org/marketing-research/choice-based-conjoint-technique.

[50] (2014). ACBC Technical Paper. https://www.sawtoothsoftware.com/support/technical-papers/adaptive-cbc-papers/acbc-technical-paper-2009.

[51] Rochon D., Eberth J.M., Fraenkel L., Volk R.J., Whitney S.N. (2014). Elderly patients’ experiences using adaptive conjoint analysis software as a decision aid for osteoarthritis of the knee. Health Expect..

[52] Stacey D., Hawker G., Dervin G., Tomek I., Cochran N., Tugwell P., O’Connor A.M. (2008). Management of Chronic Pain: Improving shared decision making in osteoarthritis. BMJ.

[53] Austin C.A., Mohottige D., Sudore R.L., Smith A.K., Hanson L.C. (2015). Tools to promote shared decision making in serious illness: A systematic review. JAMA Intern. Med..

